# Neuro-lymphaphotonics opens new horizons of the future technologies for the therapy of brain diseases

**DOI:** 10.7150/thno.120374

**Published:** 2026-01-01

**Authors:** Shaojun Liu, Oxana Semyachkina-Glushkovskaya, Tingting Yu, Egor Ilukov, Edik Rafailov, Sergey Sokolovski, Jürgen Kurths, Dan Zhu

**Affiliations:** 1 MOE Key Laboratory for Biomedical Photonics, Wuhan National Laboratory for Optoelectronics-Advanced Biomedical Imaging Facility, Huazhong University of Science and Technology, Wuhan, Hubei 430074, China.; 2 Department of Biology, Saratov State University, Astrakhanskaya 82, Saratov, Russia, 410012.; 3 Optoelectronics and Biomedical Photonics Group, AIPT, Aston University, Birmingham B4 7ET, UK.; 4 Institute of Physics, Humboldt University, Newtonstrasse 15, Berlin, 12489, Germany.; 5 Department of Complexity Science, Potsdam Institute for Climate Impact Research, Telegrafenberg A31, Potsdam, 14473, Germany.

## Abstract

Pharmacological treatment of brain diseases is hampered by the blood-brain barrier that prevents the vast majority of drugs from entering the brain. For this reason, the pharmaceutical industry is reluctant to invest in the development of new neurotropic drugs. Even if effective pharmacological strategies for the treatment of brain diseases will be found, it will take 10-15 years between the emergence of an idea and the introduction of a drug to the market. This creates priority for the development of neuro-lymphaphotonics based on the development of promising non-pharmacological strategies for managing functions of the meningeal lymphatic vessels (MLVs). MLVs play a crucial role in the removal of toxins and metabolites from brain as well as in regulation of brain homeostasis and its immunity. Since MLVs are located on the brain surface, light penetrating the skull easily reaches MLVs and affects their functions. Therefore, MLVs are an ideal target for photobiomodulation (PBM). The pioneering studies have shown that PBM of MLVs is a promising strategy for the treatment of a wide range of neuropathology, including Alzheimer's or age-related brain diseases, brain tumor, intracranial hemorrhage, brain damages caused by diabetes. It has recently been discovered that sleep enhances the therapeutic effects of PBM and is a "therapeutic window" in overcoming the limitations of PBM in the elderly. Considering that the PBM technologies are non-invasive and safe with commercially viable possibilities (portability and low cost), neuro-lymphaphotonics open up promising prospects for the development of future technologies for the effective therapy of brain diseases.

## 1. MLVs as the targets for photobiomodulation (PBM)

The meningeal lymphatic system is a transparent network of thin vessels located in the dura mater of the brain and the spinal cord around the main venous sinuses and the meningeal arteries. It is known that there are two types of the meningeal lymphatic vessels (MLVs), dorsal and ventral 1. The difference is that the dorsal MLVs have valves and are directly involved in the drainage of brain tissue, while ventral ones do not have valves and form tunnels for the passive movement of brain fluids from the central nervous system (CNS) to the periphery.

MLVs were discovered two centuries ago by the Italian anatomist Paulo Mascagni, but only in 2015 his discovery was recognized by the scientific community 2, 3. This is due to the fact that no one except Mascagni was able to detect transparent MLVs 4. It should be noted that Mascagni himself was successful only with a few samples of the brain meninges of patients who died from hydranencephaly, when MLVs were significantly enlarged due to pathology 5. Although in 1953 another Italian scientist Lecco also described MLVs in the 4 of 30 meninges, nevertheless, the lack of methods to effectively detect MLVs did not give much interest to this work 6. Only recently, thanks to advances in neuroimaging, biochemistry, and genetics, has the golden key to the door of promising opportunities for studying the functions of the lymphatic system become available, including reliable markers of the lymphatic endothelium, confocal microscopy, and transgenic mice with the fluorescent lymphatic vessels 1, 3, 7-9. This has allowed significant progress in understanding the role of MLVs in maintaining homeostasis and immunity in CNS 10-12. Currently, the field of studying the MLV functions is one of the most promising and in-demand in neuroscience.

The high interest in the investigations of the MLV functions is associated with their key role in the brain drainage and removal of toxins from its tissues, which is a necessary for the normal function of CNS. Indeed, the MLV dysfunction is associated with many brain diseases, such as Alzheimer's disease (AD) 7, 13, Parkinson's disease (PD) 14, traumatic brain injury (TBI) 15, brain cancer 16, 17, and intracranial hemorrhages 18, 19. The role of MLVs in the development of various brain diseases is well presented in more detail in the following reviews 10-12.

The age-related changes in MLVs underlie brain aging 1, 7. It is known that the MLV functions begin to decline in middle age, followed by progression in the late stages of ontogenesis 1, 7. An age-related decline in the MLV functions is accompanied by a gradual increase in the level of amyloid-beta (Aβ) and a decrease in cognitive abilities 7. With age, the lymphatic valves lose the ability to effectively control lymph flow, which compensatory leads to lymphatic hyperplasia 20, 21. These age-related morphological changes in the lymphatic endothelium are the cause of the age-related decline in the functions of the lymphatic system 1.

An intriguing event may be the discovery of the lymphatic vessels directly in the brain tissue. MLVs are not related to the brain; they are located in the meninges that cover the brain from above, but have no contact with its tissues. Therefore, even such an important event as the recognition of the presence of MLVs by the scientific community does not provide an idea of how fluids, metabolites and toxins are removed from the brain. Pilot studies indicate the discovery of lymphatic structures and signs of the lymphatic vessels in the brain of humans and mice, which probably connect with MLVs into a cerebral lymphatic network 22-25. When this fact is proven, it will change our mentality and scientific concepts about the lymphatic processes of clearance, drainage and immunity in CNS.

It is discussed in the scientific community that the development of strategies for stimulation of the MLV functions will be an advance in the treatment of brain diseases 7, 26-28. However, the lymphatic vessels have no direct contact with the blood and is involved only in the return of intercellular fluid to the venous system 29. Therefore, pharmacological drugs delivered with the blood will not have an effect on the lymphatic system. A method of introducing the vascular endothelial growth factor C into the cisterna magna for lymphangiogenesis has been proposed to improve the effects of immunotherapy for AD 13. However, this is an invasive method that is limited for its wide application in routine clinical practice. Recently, the deep cervical lymphatic-vein anastomosis surgery is gradually becoming one of the hot directions for international AD treatment 30. The experts unanimously agree that the surgical design is a shunt that reconstructs the deep cervical lymphatic system by anastomosing it with adjacent veins, thereby immediately reducing the pressure in the brain's lymphatic system and gradually improving the accumulation of metabolic proteins in the brain (such as Aβ, Tau, and α-synuclein), and improving and alleviating the symptoms of AD 31. However, these are also surgical interventions that cannot be widely applied in routine clinical practice to every patient.

Since MLVs are located on the surface of the brain, they are an ideal target for phototherapy or PBM. PBM, including photobiomodulation, is based on the action of light in the therapeutic window (600-1300 nm) 32. Passing through the scalp and skull, the light is partially scattered and significantly loses its energy, so only a small part of it reaches the brain 32, 33. Therefore, PBM is also called low-intensity laser therapy. The most popular in clinical practice for the treatment of brain diseases are lasers and light-emitting diodes (LEDs) with wavelengths above 800 nm, which penetrate deeper through the skull 32-35. There are fundamental studies that describe in detail the use of PBM in the treatment of brain diseases 36-38. However, for almost 50 years of PBM use in medicine, little is known about the direct targets for light, due to which phototherapeutic effects are achieved. It is generally accepted that light activates the mitochondrial enzyme cytochrome C oxidase and increases the production of nitric oxide (NO), which leads to an increase in the energy status of neurons and microcirculation of brain tissue 32, 35. These non-specific changes in response to light exposure provide two important mechanisms for restoring brain function, including energy and oxygen supply.

However, until now there has been no clear understanding in biophotonics of how PBM can be used to correct brain functions, improve memory, reduce neuroinflammation, and increase brain drainage. The explanation of the PBM -dependent increase in the activity of the cytochrome C oxidase (COX) activity and NO production does not allow us to answer these questions. Therefore, it remains unclear in which anatomical structures of the brain light exert therapeutic effects. In other words, there is a large gap between the well-known molecular effects of light and the systemic responses of the brain to PBM. This significantly hinders the widespread use of PBM in routine clinical practice, since the key point for therapy is to understand the target, what the treatment will be aimed at.

Recently re-discovered MLVs became such a target. There is a growing body of research showing that PBM stimulates brain drainage in mice and rats, promoting an increase in the lymphatic removal of metabolites and toxins from CNS, such as Aβ, blood, and advanced glycation end products 19, 39-43. The actively developing field of new promising photo-technologies for enhancing the MLV functions formed the basis for neuro-lymphaphotonics as a new direction in biophotonics 44. Devices for non-invasive photostimulation of MLVs are future technologies for the treatment of brain diseases.

## 2. PBM of MLVs is a new strategy for the therapy of brain diseases

Brain diseases, including neurologic, mental and cerebrovascular diseases, account over 15% of global health loss in 2021 45. Over the past 30 years, there has been a 65% increase in prevalent brain disorders, increasing from around 2.4 billion in 1990 to 4 billion in 2021 45, 46. The associated burden is expected to increase in the coming years, creating new challenges for health systems, employers, patients, and families 45-47. The reason of this is the blood-brain barrier (BBB), which is a semi-permeable barrier encompassing cerebral microvasculature. The intact BBB impedes the influx of most blood-borne substances from entering the brain. At the same time, BBB also excludes more than 98% of therapeutics from access to the brain 48-50. Furthermore, the development of new drugs is a long, costly, and high-risk process that takes over 10-15 years with an average cost of over $1-2 billion for each new drug to be approved for clinical use 51, 52. It is also worth noting that 90% of new clinical drug development fails 52. Therefore, the development of innovative non-pharmacological methods of treating brain diseases is a priority task of modern medicine.

PBM of MLVs opens up new perspectives for the development of promising and breakthrough strategies for the therapy of brain diseases. The first works in this direction were done using a new generation 1267 nm laser 19, 39, 41, 53, 54. Pioneering studies have shown that the 1267 nm radiation stimulates the MLV functions increasing their contractility and drainage properties 19. This facilitates lymphatic removal of metabolites and toxins dissolved in the cerebral spinal fluid (CSF) from CNS to the cervical lymph nodes, which are the first anatomical station for collection of CSF 3. Based on these photo-effects, clinically significant results have been obtained in various studies. Indeed, 1267 nm-stimulation of lymphatic removal of blood provides better recovery from intracranial hemorrhages 19. The 1267 nm-activation of brain drainage contributes to increase in resistance to glioblastoma progression and microglia injury caused by diabetes mellitus 39, 55. The PBM of MLVs also stimulates lymphatic clearance of Aβ, which improves cognitive function in mice with AD 40, 41. Recently, it has also been shown that PBM with an 880 nm laser significantly improves cognition of transgenic mice with AD (5xFAD and APP/PS1) leading to reducing Aβ deposition, neuroinflammation and neuronal damage 56.

Modulation of brain drainage and clearance is regarded as a promising direction in the emergence of breakthrough technologies for the treatment of brain diseases 57-59. In this aspect, the non-invasive PBM technologies have great advantages to take a competitive position compared to the pharmacological strategies for modulating the MLV functions. Indeed, the PBM methods are recognized by the U.S. Food and Drug Administration (FDA) as safe and can be used even at home, which is important for patients who require a long-term therapy. The PBM devices, especially those that include LEDs, do not require a complex equipment for their production, PBM components have a commercially advantageous price, which creates conditions for their rapid entry into the medical equipment market and implementation in routine clinical practice.

## 3. Direct generation of singlet oxygen triggers the MLV activation: future perspectives for clinical application

The most promising wavelengths (laser 1267 nm, LEDs 1050 nm) for photostimulation of MLVs are those that are capable of generating singlet oxygen (^1^O_2_) directly in living tissues 40, 44, 60. Indeed, ^1^О_2_ can be generated directly in living tissues with light at 1270 nm, 1064 nm and other wavelengths 44, 60. The first works in physiological application of direct ^1^O_2_ generation were focused exclusively on cellular studies of the ^1^О_2_ effectiveness of killing of tumor cells 61, 62. This was largely due to the limited targets whose functions could be modulated using a direct generation of ^1^О_2_ in the body, where the concentration of ^1^О_2_ is obviously less than in the isolated cells in *in vitro* experiments. This is due to the significant scattering of radiation energy when passing through the skin, especially through the skull. In this sense, there were doubts in the scientific community that direct ^1^О_2_ generation would find wide clinical applications.

The discovery of MLVs as the targets for the physiological effects of ^1^О_2_ have strongly changed the situation and opened up promising strategies for the clinical application of photo-technologies for direct ^1^О_2_ generation in the meninges 44. Indeed, the high efficiency of using PBM with both a 1267 nm laser and 1050 nm LEDs, i.e. with wavelengths that generate ^1^O_2_ in tissues 44, 60, was shown for photo-activation of brain drainage and lymphatic removal of metabolites and toxic products 19, 39-43.

**Figure 1:**
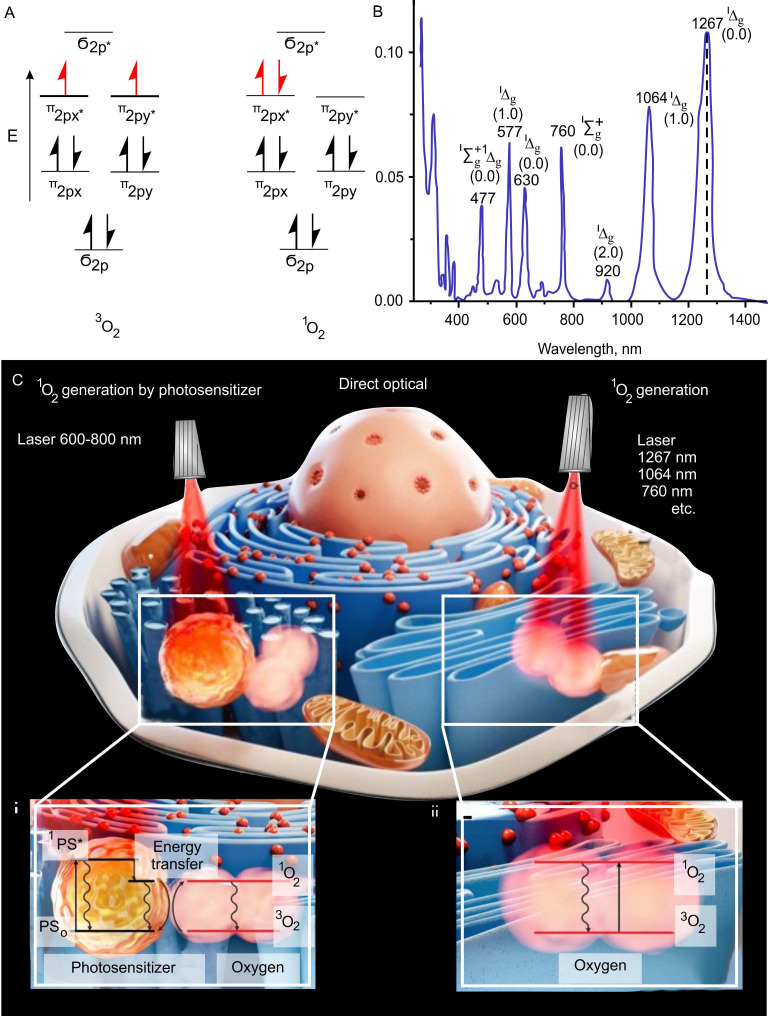
Mechanisms of ^1^O_2_ generation. (A-B) Triplet and singlet states (A) and absorption bands (B) shows highest peak absorption at 1267nm. Adapted with permission from 65 (Copyright 2018 IntechOpen) and 66 (Copyright 2023 Royal Society of Chemistry). (C) Two main mechanisms of ^1^O_2_ generation. Left side - photodynamic mechanism of ^1^O_2_ generation by photosensitizer through energy transfer to oxygen from singlet and triplet exited states of photosensitizer (^1^PS* and ^3^PS*) and right side - direct optical generation of ^1^O_2_. 1267 nm, 1064 nm and 760 nm are the maxima of light absorption by molecular oxygen. Adapted with permission from 73, Copyright 2022 Optica Publishing Group.

The basic triplet state of oxygen has several absorption bands in the visible and infrared regions (optical range between 390 nm to 1300 nm), at which ^1^O_2_ can be produced 63 (Figure 1A-C). A recent study has shown the ability of 1267 nm light to directly generate ^1^O_2_ at the highest absorption peak of triplet oxygen and that direct laser generation (photosensitizer-free) of certain concentrations of ^1^O_2_ can stimulate mitochondria bioenergetics in normal astrocytes and neurons without inducing cell death 64. Thus, certain doses of ^1^O_2_ work as an activator of cell mitochondrial respiration and the adenosine triphosphate (ATP) production.

A triplet state occurs in cases where the spins of two unpaired electrons, each having spin s = 1/2, align to give S = 1 (ground state), in contrast to the more common case of two electrons aligning oppositely to give S = 0, a spin singlet (excited sate) (Figure 1A).

^1^O_2_ is a common name for the electronically excited state of triplet oxygen (Figure 1B). The ^1^O_2_ is formed when the spin of one of the electrons located on different π-antibonding orbitals of the oxygen molecule changes. Molecular oxygen has two low-lying singlet excited states above the triplet state 65, 66. These states differ, in addition to differences in electronic configurations, in energy and lifetime. The reactivity of the excited state is several orders of magnitude larger than that of the triplet form of oxygen, which, due to its biradical chemical character, is unreactive to most chemical compounds 67. The ^1^O_2_ exhibits considerable reactivity toward electron-rich organic compounds, especially, lipids, proteins, nucleic acids and ribonucleic acid 68, 69. This leads to the formation of such reactive substances as endoperoxides, radical oxygen species, peroxides, aldehydes, etc.

The appearance of data on the participation of ^1^O_2_ in the regulation of physiological functions of cells and the possibility of its activation with the use of photosensitizers have made a significant step towards understanding the role of this highly reactive type of oxygen as the main mediator of therapeutic effects in photodynamic therapy (PDT) 70, 71. The photosensitized generation of ^1^O_2_ requires only oxygen, light of an appropriate wavelength, and a photosensitizer capable of absorbing and using that energy to excite oxygen to its singlet state (Figure 1C-i) 72, 73.

The ^1^O_2_ oxygen is produced via an energy transfer during the collision of an excited sensitizer with triplet oxygen. The excitation of the sensitizer is usually achieved by using a one-photon transition between the ground state (PS_0_) and the singlet excited state (^1^PS*) after light illumination in the of visible or near-infrared (NIR) spectral range. Intersystem crossing generates the triplet state of the sensitizer (^3^PS*) with a longer lifetime than that of ^1^PS*. In the ^3^PS* state, the sensitizer reacts with the oxygen molecule. Sensitizers can accumulate in various cell compartments 74. So, the effectiveness of PDT varies depending on the characteristics of selective accumulation of a sensitizer in target tissues.

The possibility of a direct excitation of an oxygen molecule by light in the ground triplet state and regulation of its production by changing the light intensity and exposure time is of undoubted interest for fundamental and practical medicine. The basic triplet state of oxygen has several absorption bands at which ^1^O_2_ can be produced (Figure 1C-ii) 75. Photon absorption in the particular absorption wavelengths corresponding to different electronic-vibrational molecular levels leads to the excitation of the specific ^1^O_2_ state. A single photon can generate one or two ^1^O_2_ molecules, and thus the monomol and dimol transitions are realized. The 1267 nm, 1064 nm and 760 nm bands are most widely used to directly generate ^1^O_2_ 76, 77.

**Figure 2:**
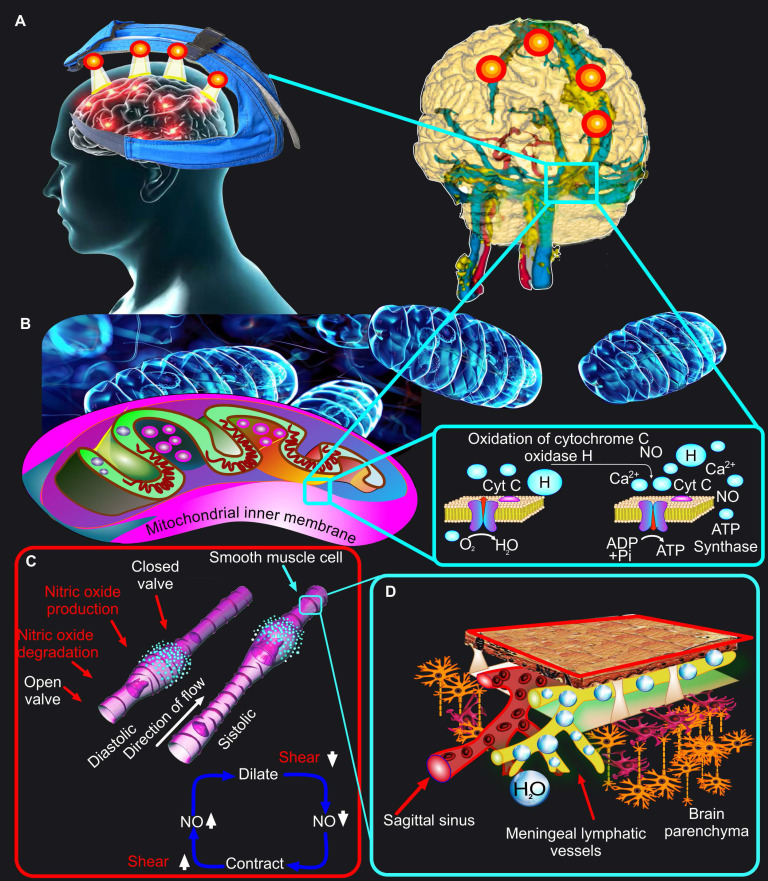
Mechanisms of the PBM effects on MLVs. (A) Schematic representation of the world's first device for PBM of MLVs in humans. (B) Depiction of a widely accepted concept of the PBM mechanism based on PBM-mediated oxidation of the cytochrome C oxidase. (C) The NO-dependent mechanism of PBM effects on MLVs is based on the stimulation of peristaltic processes in the lymphatic vessels. (D) PBM-mediated stimulation of the peristaltic process in MLVs promotes their drainage and cleansing functions.

However, light sources emitting around 1267 nm are scarce and expensive, which makes them commercially unattractive. In this regard, light irradiation 1064 nm is much more perspective due to the coincidence of this band with the light emitted by 1267 nm lasers 44, 60. Despite the fact that for any biomedical purpose excitation at 1064 nm is equal to that at 1267 nm, there are two main fundamental and practical advantages for 1064 nm 60. The 1064 nm irradiation has a 10-fold reduction in water absorption as compared to 1267 nm, which allows 1064 nm to save more therapeutic energy. The 1064 nm absorption band coincides with the emission from commercially available lasers (~1064 nm) and LEDs (~1050 nm). Since LEDs are widely used in clinical practice for PBM and are recognized by the FDA as safe technologies, as well as due to their commercially attractive price, they are the most promising for their implementation in clinical practice. Indeed, the first studies in this direction indicate the potential for using LED 1050 nm to effectively remove toxic Aβ from CNS in order to improve cognitive functions of the brain 42, 43. Recent *in vivo* studies on mice using LEDs have shown that among the wavelengths of 880 nm, 900 nm, 1050 nm and 1300 nm, the light 1050 nm has the greatest stimulating efficacy in pulsed mode on the MLV functions and lymphatic removal of Aβ 43. It is noteworthy that the world's first device for PBM of MLVs in humans is based on LEDs 1050 nm, which is undergoing clinical trials in 2025-2026 [The number of registration is 17491 from 27.02.2025, https://reszdravnadzor.gov.ru] (Figure 2A).

## 4. Sleep increases the PBM therapeutic effects

The use of PBM during sleep as a new direction in biophotonics emerged only 5 years ago, but very quickly received a great resonance. Despite the obvious facts that sleep is the best medicine for many diseases, nocturnal therapy and especially photo technologies for this do not yet exist. In 2020, the idea of photo-therapy of AD during sleep was first published 78. This work stimulated the development of new approaches in this direction 79, 80. In 2023, the idea of developing innovative technologies for phototherapy during sleep was proposed 28. In this work, it was clearly shown that there are transcranial phototherapy devices on the commercial market, but all of them are not suitable for use during sleep, because they are large, heavy, noisy and uncomfortable in the form of helmets or hats. On the other hand, sleep monitoring devices are portable, presented in the form of rings, bracelets and smart watches. However, there are no solutions for their joint operation with PBM devices yet. Since the use of PBM in sleep is a pioneering direction, there are no data about the impact of different circadian rhythm changes on the action of PBM yet. However, as discussed in the section 6, it is not so much the circadian stages that are important for PBM, but rather the changes in the brain's glymphatic and lymphatic drainage pathways associated with deep sleep.

Modern trends in the development of photo-technologies are aimed at creating portable, safe and easy-to-use devices that can be used at home, in a car, or on an airplane, not only in a hospital. In this direction, technologies for PBM of MLVs occupy a leading position. The world's first device for PBM of the MLV functions during sleep has been created (Figure 2A). The development of this technology is based on preclinical results indicating a significant increase in the therapeutic effects of PBM during sleep. Indeed, photostimulation of a lymphatic clearance of Aβ from the brain of mice is stronger during deep sleep than during wakefulness, which also contributes to a better improvement of the neurocognitive status in animals that received PBM under EEG-control of sleep 40, 81. The background for the idea of PBM of MLVs during sleep arose from the discovery of the fact of activation of drainage in sleeping brain. In 2013, original results were published on mice using multiphoton microscopy, which revealed an increase in perivascular spaces in brain tissues during deep sleep. This contributes to a better exchange between the blood and the brain, as well as the removal of metabolites 82. Later, in 2019, the data were obtained on humans using functional magnetic resonance imaging (fMRI), which clearly showed an increase in CSF oscillations during deep sleep 83. Subsequent studies in this area have found that deep sleep is accompanied by activation of lymphatic removal of toxic metabolites from the brain 40, 43, 81. At the same time, the destruction of MLVs is also restored faster when using PBM during deep sleep 40. Interestingly, the use of PBM during sleep significantly increases the learning ability in healthy mice than during wakefulness 42.

The direction of PBM application during sleep is pioneering, therefore additional research is required for a better understanding of the mechanisms underlying this phenomenon. However, it is obvious that the best therapeutic effects of PBM during deep sleep are associated with the natural activation of the process of brain drainage and clearance of metabolites, which ensures the maintenance of immunity and homeostasis of CNS 27, 28, 78, 84.

## 5. Mechanisms of the PBM effects on the endothelium of the blood and lymphatic vessels

It is a generally accepted fact that the physiological effects of PBM are based on oxidation of COX and an increase in tissue microcirculation due to stimulation of NO production in the endothelium of blood vessels 85 (Figure 2B-C). Traditionally, it is believed that NO only relaxes the blood or lymphatic endothelium. Indeed, in relation to the endothelium of the blood vessels, it is well known that PBM increases brain microcirculation, thereby exerting therapeutic effects in TBI, AD and other neurological damages 86, 87. However, from the standpoint of the vascular physiology, and especially the physiology of the lymphatic vessels, not everything is so simple. NO is actively involved in the process of regulation of the contractility of the lymphatic vessels 88, 89. There is a hypothesis based on experimental data that photo-effect on the endothelium of basal MLVs leads to the NO formation, mainly in the valves because 50% of the endothelial NO-synthase is localized there 19, 88. The release of NO stimulates the dilation of MLVs and increases their permeability, which leads to an increase in their volume due to the influx of fluid into them. At this moment, the upstream valve is open, but the downstream valve is closed. When MLVs are filled, share stress decreases and NO is degraded. Afterward, a subsequent contraction of MLVs is initiated through the Ca^2+^ influx both via stretch- voltage-, or ion-activated channels and from the depot. The contraction of MLVs closes the upstream valves and opens the downstream ones leading to an increase in wall shear stress and the NO production locally, thus starting the cycle again. This way is the peristaltic process in MLVs, which is the basis of their drainage and cleansing functions (Figure 2C).

Thus, PBM (1050 nm and 1267 nm) stimulates the MLV functions via NO-pathway. It has recently been shown that two wavelengths of lasers (1064 and 1270 nm) induce NO release in cultured human endothelial cells but not in neurons 90. PBM (1064 and 1270 nm)-induced NO release is accompanied by phosphorylation of the endothelial nitric oxidase (eNOS) that is abolished by inhibiting mitochondrial respiration, including protein kinase B (Akt) pathway 90. Other inhibitors of Akt activation pathways, such as a specific inhibitor of PI3K (Wortmannin), Src family (PP1), PKC (Gö6983), do not affect this response 66. These findings suggest that the Akt activation caused by PBM involves mitochondrial retrograde signaling. Thus, PBM (1064 and 1270 nm) induces the generation of NO in endothelial cells via Akt and eNOS phosphorylation.

Previously, using lasers of other wavelengths (630-900 nm), it was shown that PBM can increase NO bioavailability in living tissues via other pathways. So, PBM stimulates photo-dissociate NO from COX in mitochondria 91, 92. Indeed, NO is a reversible inhibitor of COX 93 because NO can bind to heme a3 in competition with oxygen leading to formation of reactive oxygen species (ROS) 94, 95 and reducing oxygen consumption by inhibition of COX what is the basis of activating ROS- or NO-mediated signaling 96, 97. For example, on the cardiac ischemic injury model has been shown that the cytoprotective effects of PBM (670 nm) are mediated through an increase in the ATP activity via NO dissociation 98. PBM (590 nm, 660 nm) also can increase NO bioavailability from intracellular stores, including from pre-existing nitrite tissue stores 99, 100, hemoglobin, nitrosyl hemoglobin and nitrosyl myoglobin 101.

Hence, the physiological effects of PBM are mediated not only by the direct effect of light on oxygen metabolism in neuronal cells, but also are through an increased blood/lymph flow via augmented NO generation in the endothelial cells. Notably, there are clinical studies of PBM (1064 nm laser), which clearly demonstrate a significant increase in cerebral blood oxygenation in humans 102-104. Remarkably, PBM (1267 nm laser, 1050 nm LEDs) stimulates the drainage and clearing function of MLVs and alleviated neurocognitive deficits associated with the accumulation of Aβ in mice or the presence of the blood in the brain after intraventricular hemorrhage 19, 40, 41, 43. These results collectively suggest that the beneficial effects of PBM on the cerebral drainage system 105 are also mediated by NO in the lymphatic endothelial cells.

There are several other mechanisms by which a PBM-mediated increase NO production can modulate the lymphatic tone and contractility: 1) the activation of an iron-regulatory factor in macrophages 106, 2) the modulation of proteins such as ribonucleotide reductase and aconitase 107, 108; the stimulation of the adenosine diphosphate (ADP)-ribosylation of glyceraldehyde-3-phosphate dehydrogenase and protein-sulfhydryl-group nitrosylation 109, 110. PBM (1267 nm) causes an increase in the permeability of lymphatic walls and a decrease in the expression of tight junction proteins 111.

Although the precise mechanisms of the PBM effects on MLVs are still elusive, recent studies consistently report that PBM affects the tone and permeability of MLVs facilitating brain drainage and lymphatic removal of metabolites and toxins from CNS, which alleviates brain pathologies 19, 41-43, 53, 54.

## 6. Glymphatic/lymphatic mechanisms of the PBM effects: from drainage to brain regeneration and cognitive functions

Since brain drainage and MLVs are considered as important targets for PBM 26, 40, 78, it is obvious to expect that the mechanisms of therapeutic effects of PBM are based on photostimulation of brain drainage and the closely related processes of glymphatic/lymphatic removal of metabolites and toxins dissolved in CSF from CNS. Indeed, a number of experimental studies have shown that PBM-mediated stimulation of MLVs promotes lymphatic removal of Aβ from the brain, which is accompanied by an improvement in the cognitive functions of animals 40, 41, 81. Similar results were obtained in a model of intraventricular hemorrhage 19. It was clearly shown that PBM increases the removal of blood from the brain via MLVs into the deep cervical lymph nodes, which significantly improves the recovery of mice after hemorrhagic event 19.

How PBM can improve cognitive function remains poorly explored. Growing evidence show a crucial role of brain drainage in maintaining cognitive function 7, 13, 81, 112. Indeed, the MLV dysfunction results both in reducing brain drainage and in cognitive alterations 7, 19, 40-43, while the increase in lymphangiogenesis of MLVs significantly improves the drainage of macromolecules and cognitive functions 7, 13. Thus, dysfunction of brain drainage might provide an important contribution to neuro-degenerative diseases 7, 13, 112.

How brain drainage and cognitive function are related? There are several striking examples on mice of different ages, including old animals, using the Pavlov method of forming conditioned reflexes, when PBM simultaneously increases the rate of formation of conditioned reflexes and improves brain drainage 54, 113, 114. Brain drainage, providing cleansing of its tissues due to the movement of fluids, thereby contributes to the improvement of memory formation as a fundamental cognitive function that is essential for learning and logics. Indeed, the formation of memory is based on the formation of new synaptic contacts, the movement of neurotransmitters and a whole complex of biochemical processes at the level of synaptic contacts, where brain fluids are also present and the renewal of which is a necessary process in the transmission of signals in chemical synapses 115, 116.

Conditioned reflexes are formed by activating a large number of nerve centers, including motivation (hunger and interest), memory (especially, the formation of memory in the hippocampus playing a crucial role for forming, consolidating, and retrieving memory as well as in transferring information from short-term to long-term memory during training process), and emotions (positive reinforcement in the form of food reward) 117. The transition short-memory to long-term memory known as memory consolidation that is a biological process where transient neural activity patterns become more stable and enduring, forming lasting memories. In Pavlov's conditioning, repeated pairings of conditioned and unconditioned stimulus strengthen the connections between neurons involved in the memory trace. This involves changes in the structure and function of synapses, the connections between neurons. The hippocampus plays a crucial role in the initial stages of memory formation and the conditioned reflexes. It helps retrieve information from working memory and begin the process of establishing new neural connections. The formation of long-term memories is not instantaneous. It involves a time-dependent process where detailed memories can be transformed into more generalized or semantic representations over time 118, 119. The emotionally significant events are an important factor in the formation of conditioned reflexes, where a neutral stimulus becomes a conditioned stimulus, eliciting a conditioned emotional response. Strong emotional responses influence the consolidation process, potentially strengthening or weakening memory depending on the context and intensity 120. Sleep plays a critical role in memory consolidation, allowing the brain to further strengthen and reorganize neural connections 121.

Thus, PBM by stimulation of the movement of brain fluids, clears the space for biochemical processes underlying the formation or improvement of memory as a major process of cognitive function, which may explain the dual effect of PBM on improving cognitive abilities and brain drainage as well as their physiological interaction.

An important issue is the fact that PBM has stimulating effects on MLVs located on the brain surface. However, the therapeutic effects of PBM, as noted above, affect the entire brain, including the interaction of various centers, which underlies the formation or improvement of memory and cognitive function. The explanation lies in the mechanisms of brain drainage, which is a system of interacting spaces filled with brain fluids, including CSF and interstitial fluid (ISF). Since CSF is formed daily (350 µL/min in humans and 0.32 μL/min in mice) 122 in the ventricles of the brain, CSF exits the ventricles into the subarachnoid space and then flows into the perivascular spaces (PVSs), where exchange between the blood vessels and the brain occurs. Nutrients enter from the blood vessels through BBB, are transported either by diffusion or by carriers in PVSs filled with the CSF flow and then with ISF enter the brain tissue 123. At that time, it is believed that it is in deep sleep, due to the expansion of PVS 82, that the brain tissues give up nutrient compounds to PVSs and with the CSF flow, unnecessary metabolites enter the subarachnoid space, where they either enter the venous blood through the arachnoid villi or enter MLVs and are carried out to the cervical lymph nodes, which are the first anatomical collection station for CSF flowing from the brain 1, 3, 7.

The glymphatic hypothesis, which appeared several years ago, can partly explain the PBM-mediated removal of metabolites from the brain, for example, Aβ through PVSs of cerebral arteries and veins 124-126. However, at present, due to technical limitations in studying fluid movement in the brain, it is difficult to answer the question of what mechanisms (glymphatic, lymphatic, or other) underlie the PBM-stimulating effects on brain drainage. On the one hand, there are data clearly indicating that during deep sleep, brain drainage increases due to the expansion of the size of PVSs and changes in the volume of astrocytes, which creates special spaces for the movement of brain fluids 82, 83, 127. However, for the targeted movement of fluids in the brain, a system of special vessels must exist that will direct this movement in a certain direction. Despite the 100-year history of studying brain drainage and the known pathways for this, such as PVSs 128-130, the lymphatic vessels in the ethmoid bone and along the exit of the nerves from the brain 128, 131, as well as the obvious fact that the cervical lymph nodes are the first anatomical collection station for CSF from the brain 1, 3, 7, the mechanisms of metabolite removal from CNS remain unclear. The proposed glymphatic hypothesis, in which the aquaporin channels play an important role in a driving force and arterial pulsation 124-126, nevertheless contains many unresolved critical physiological issues that are actively discussed by neurophysiologists 128, 132-134. In addition, the fact of glymphatic flow has not been experimentally proven. A similar situation is with the lymphatic vessels, the existence of which in the brain has also not been proven 22-25. There are original results that demonstrate circadian changes in the activity of the glymphatic and lymphatic systems of the brain 135. However, most likely, these two drainage mechanisms work inseparably, since the obvious and indisputable facts remain that brain fluids move along PVSs and are excreted into the peripheral lymphatic system. It is important to note that the movement of tracers in the brain directly depends on their nature. Therefore, results studying brain drainage using neutral dyes or only one type of protein, such as Aβ, may not provide a complete picture explaining the mechanisms of brain drainage, especially the pathways for removing metabolites from CNS. Until the cerebral lymphatic vessels or glymphatic fluid flow are discovered, this question will not be answered unambiguously.

There are pilot results clearly demonstrating that the effects of PBM (better during sleep than wakefulness) associated with an improvement of cognitive function in old mice are realized through both perivascular and lymphatic pathways of brain drainage 114.

## 7. fMRI as a promising method for the analysis of lymph flow in MLVs

The analysis of the MLV functions and brain drainage in animal experiments usually involves the introduction of tracers of various natures, either into CSF or directly into brain tissue. However, the introduction of even contrast compounds into CSF in humans is impossible. Therefore, non-invasive methods for assessing the MLV functions are limited and require the development of new progressive solutions.

Currently, fMRI is a promising method for both visualizing the anatomy of MLVs and assessing lymph flow. The first work in this direction was published by Absinta et al. in 2017 136. These authors, using the high-resolution 3T MRI sequences and intravenous injection of gadolinium-based contrast agents (GBCAs), demonstrated similar location of MLVs along the main venous sinuses of the brain in 5 healthy volunteers and marmosets 136. Later, new approaches for non-contrast MRI imaging of MLVs based on the analysis of brain fluid flow were proposed 137-140. In 2018-2020, Kuo et al. 137, Naganawa et al. 138 and Scholkmann 139 confirmed the findings of Absinta et al. 136 by showing that a 3D-real inversion recovery MRI sequence is able to detect MLVs in humans. Kuo et al. 164 clearly demonstrate that the lymph flow in MLVs along the sagittal sinus runs countercurrent to the venous blood flow. All of these studies based on MRI imaging of MLVs without contrast agents used a well-known time-of-flight (TOF) angiography for the analysis of blood flow in vessels 140. The main principle of TOF is that the fluid flowing from outside to the image section will have greater MRI signal intensity and thus appear “brighter" than the relatively saturated protons in the imaged section 140. Usually, to measure the direction of fluid flow, in the TOF sequences is added saturation bands placed parallel on either side of the image section. The protons in the fluids flowing into the image section from the same direction of the saturation band side will be saturated before entering the image section and therefore will have a low MRI signal, while protons coming in fluids from the opposite side of the saturation band will not be saturated and, therefore they are “brighter.”

In 2022, Albayram et al. using 3D T2-fluid attenuated inversion recovery MRI relies on internal signals of protein, described direct connections between MLVs, the lymphatic vessels located along the cranial nerves and the cervical lymphatics 141. They also reported the reduced lymphatic outflow from the aging brain that was established by atrophy of cervical lymph node and thickening of the cervical lymphatic vessels.

In 2023, Kim et al. first developed the alternate ascending/descending directional navigation called as the Aladdin algorithm for an inter-slice blood perfusion MRI to estimate lymph flow in MLVs in real physiological units (mm/sec) 142. Using phantoms, the authors showed that MRI has limitations in both visualization of MLVs (less than 1 mm are not detected) and in the velocity of lymph flow (from 1 to 5 mm/sec). In this work, for the first time, it was established that the lymph flow in the dorsal MLVs, i.e. along the sagittal sinus, in humans ranged between 2.2 and 2.7 mm/sec.

Taoka et al. also discussed in their review the MRI methods with gadolinium-based contrast agents for evaluating glymphatic system and neurofluid dynamics in humans 143. These methods are based on intrathecally injection of GBCAs as tracers for diagnostic purposes. The GBCAs penetrate to the perivascular spaces of the deep brain regions from the subarachnoid space that is similar to the glymphatic pathway 144-146. They argue that these findings support the glymphatic system hypothesis and suggest that GBCAs can be used to evaluate system activity.

## 8. Optical clearing methods for imaging MLVs and meningeal lymphatic drainage

Optical imaging plays an important role in the analysis of structure and function of MLVs in high resolution. However, most optical imaging techniques are suffered from limited light penetration due to the high scattering of tissues. The rise of the tissue optical clearing technique has provided new perspective for deep-tissue imaging by altering the optical properties of tissues to improve light penetration with various chemical and physical approaches. Various methods have emerged in the past two decades, involving ex vivo and in vivo methods, and have facilitated the advancements of life science by providing insights in deep tissues^147^, ^148^.

Indeed, the ex vivo clearing methods have been widely used in 3D visualization of whole organs and even whole bodies by combining with the light sheet microscopy. Li et al. utilized the iDISCO^+^ clearing method to visualize the Aβ density of the whole brain and reported the difference of PBM-mediated stimulation of Aβ clearance in different brain region of 5xFAD mice 41. Some study reported the applications for the MLVs, which located between the skull and the brain. For instance, Cai et al. developed the vDISCO method for panoptic imaging to address the risk of destroying of the connections when the brain or the meninges were harvested for standard histology, and readily observed the previously described the lymphatic vessels along the sagittal sinus, the pterygopalatine artery and the transverse sinus in the Prox1-EGFP reporter mice 149. Mai et al. utilized the wildDISCO to achieve whole-body immunolabeling and showed the lymphoid elements positive (LYVE-1^+^) vessels connecting the olfactory bulb with the cortex, and observed LYVE-1^+^ and podoplanin positive vessels entering the brain parenchyma around thalamus 150. The recently published SOLID clearing method 151 can further minimize the tissue distortion of brain-wide profiling of diverse structures, providing a potential alternative for mapping of the anatomy of MLVs and the brain drainage system. Meantime, Chang et al. reported the existence of the lymphatic vessels in the brain parenchyma and characterized their features in different brain regions with tissue clearing methods. They combined the iDISCO^+^ clearing method, LYVE-1 immunostaining and light sheet microscopy to clear, label and image the whole brain, and detected the LYVE-1 positive signals on the brain meninges, as well as deep areas of the brain, vessel-like structures were observed in the cortex, cerebellum, hippocampus, midbrain, and brainstem, and further confirmed the observations with CLARITY method in thick brain sections 24. However, the existence of the lymphatic vessels in the brain parenchyma is still controversial. Siret et al. exclude the presence of “bona fide” lymphatic endothelial cells within the brain parenchyma using multiple lymphatic reporter models 152. A recent work by Li et al. also reported that the slc6a11b+ RAs cooperate with calcium-binding EGF domain 1 (CCBE1)+ fibroblasts to restrict muLEC growth on the brain surface via controlling mature VEGF-C distribution, hence explaining why the meningeal mural lymphatic endothelial cells do not invade the brain parenchyma 153.

Except for the overall architecture of brain lymphatic vessels, the anatomic drainage routes of brain lymph fluid (BLF) had been less explored since the limitation of classic immunohistological techniques. He et al. made efforts to dissect the anatomy of the BLF pathway in a rat model by injection of Evans blue to lateral ventricle 154, the bright-field observation is unable to obtain the exact 3D anatomic drainage routes. Benefiting from the capability of tissue clearing in preserving connections between the meninges and the collecting lymph nodes, Jacob et al. performed a 3D imaging of decalcified and iDISCO^+^-cleared whole mouse head, they observed a conserved 3D anatomy of MLVs that aligned with dural venous sinuses but not with nasal CSF outflow, more importantly, they discovered the extended anterior MLV network around the cavernous sinus at the base of skull, which connects the glymphatic system and mLVs 155. Recently, Yooh et al. also identified the nasopharyngeal lymphatic plexus (NPLP) as a major hub for CSF drainage with the aid of tissue clearing. They utilized the CUBIC-L for clearing, combining with the EDTA decalcification and d-PROTOS for refractive index matching 156.

For *in vivo* optical imaging of the brain, an observation window is usually required. The in vivo skull optical clearing concept proposed by Zhu's group 157 offers a non-invasive optical window, eliminating the need of the craniotomy or skull-thinning, which may damage the dura mater or brain tissue. The successive development of SOCW 158, USOCA 159, VNSOCA 160, and TIS window 161, LCCW 162 have significantly enhanced the capabilities of different imaging modalities such as laser speckle contrast imaging, hyperspectral imaging, and multi-photon microscopy, which are commonly used for monitoring the activity and structure of cortical neurons, microglia, blood vessels, as well as some immune cells^163^, ^164^. Liu et al. combined the skull optical clearing window with two-photon microscopy to study the influence of transcranial PBM to the microglia reactivity of the diabetic mice 39. Empirically speaking, it might be difficult to apply these skull optical clearing agents directly on the observation of MLVs adjacent to the venous sinuses due to cranial sutures will allow the agents leak beneath and come into direct contact with the dura or brain tissues. Some recently reported in vivo clearing agents, such as the tartrazine 165, 166 or Iodixanol 167, may pose the potential to be applied to address this limitation with certain optimization. Recent work by Vera Quesada et al. indicated a more extensive distribution of the meningeal lymphatic vessels throughout the dura mater 168, which the skull optical clearing window will play its significant role on non-invasive monitoring.

Recently, Yang et al. reported a stereoscopic wide-field photoacoustic microscopy for intravital imaging to distinguish the morphology and MLVs and the cerebral vessels with the imaging depth of 3.75 mm 169. As we know, the performance of photoacoustic microscopy also degrades due to the tissue turbidity. As we described previously 170 the SOCS could enhance both the transmittance of light and ultrasound in the skull and elevated the photoacoustic signal. It is expected to use the in vivo clearing agent to increase the imaging quality and imaging depth of the above-mentioned photoacoustic microscopy.

## 8. Future technologies for PBM of MLVs

The diversity of PMB instruments is enormous 171. The application of PBM can be localized to various anatomical regions, including the skin 172, eyes 173, brain 171, muscles 174 and even the entire body 175. It may be necessary to utilize various hardware design specific to particular application. For instance, the most common hardware implementations of PBM devices for brain are laser handpiece 176, LED clusters 177, LED helmets 178; for eyes are glasses and lenses 173, 179; for the skin are laser handpiece and LED clusters 180, 181.

Currently, home-use PBM applications are becoming more popular 139, 182. This type of device could be bought over the counter and differs from professional medical PBM devices in its ease of use and optical and electrical safety 171, 183. In case of home-use the LED systems appear to offer a superior alternative to laser systems 171, 183.

**Figure 3:**
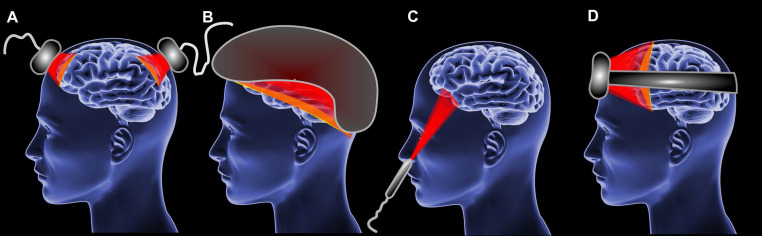
The types of PBM devices for brain. (A) LED clusters. (B) Helmet. (C) Intranasal LED. (D) Headband.

The implementation of home-use PBM devices could be different such as LED clusters (Figure 3A), helmets (Figure 3B), intranasal LED (Figure 3C) headbands (Figure 3D) 184-186. However, PBM during sleep impose additional requirements to patient convenience 28. The headband approach represents the optimal sleep-compatible design.

The design of PBM headbands appears to be more focused on the forehead and it cannot be used for PBM of MLVs. It is therefore necessary to develop a PBM sleep-compatible technology which is capable for the stimulation on the cerebral sinus of the lymphatic system of the brain.

**Figure 4:**
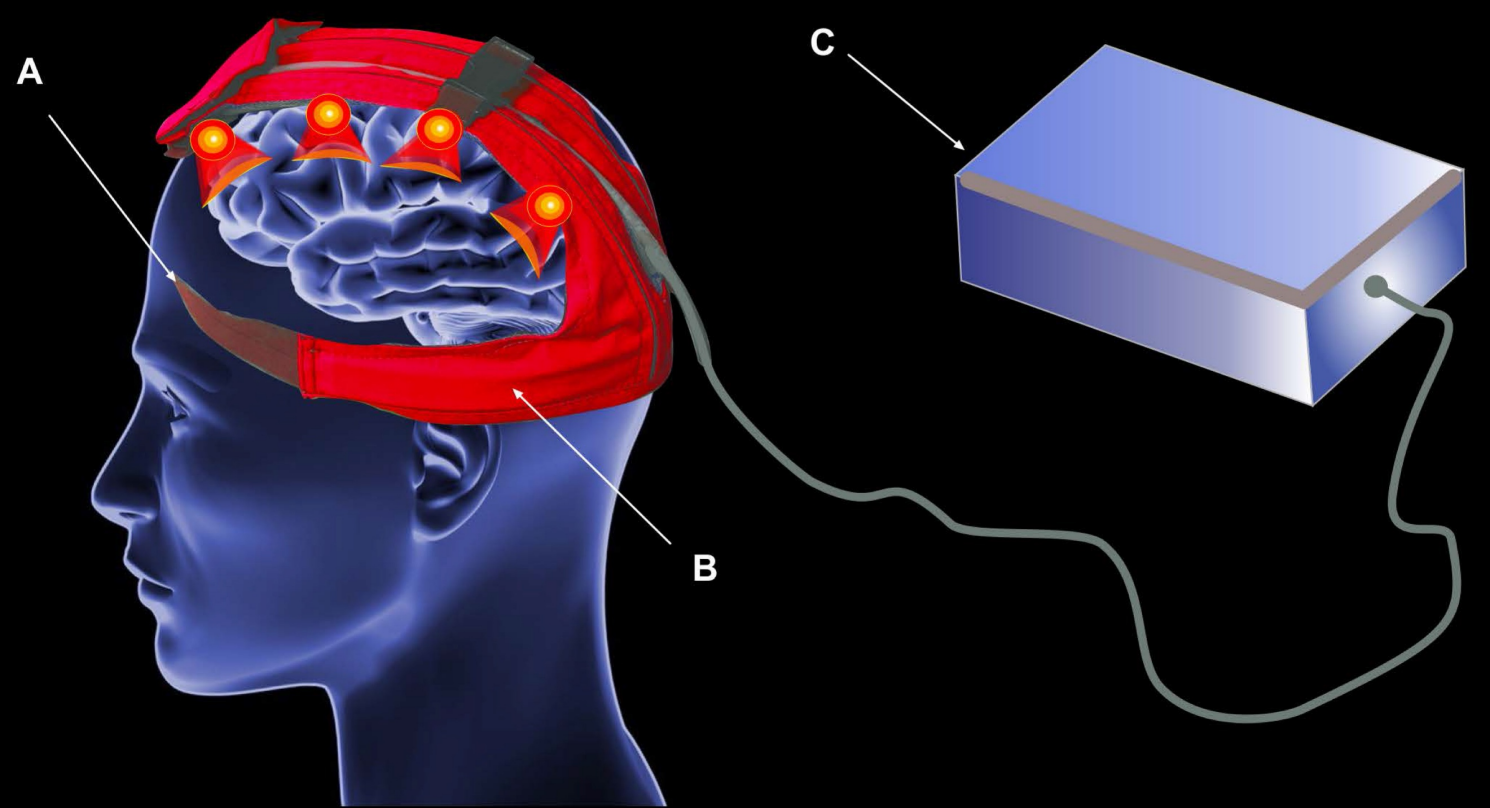
The flexible LED PCB approach, including headband (A), flexible LED PCB (B), and control module (C).

At present, there are no technologies that are specifically designed to stimulate function of MLVs. One potential approach is the use of a flexible emission element based on LEDs. This has the advantage of enabling the photostimulation to be targeted to the area of MLVs without any discomfort for patients which makes it sleep-compatible. Figure 4 shows a principal design of this approach. The LED flexible printed circuit board (PCB) is located along MLVs, the headband is used to hold the LED strips, the control module is located outside the headset which gives an ability to power the whole systems either from the large capacity battery or from mains power.

However, this approach has also disadvantages, primarily due to the snug fit of LEDs to the head. The inability to achieve uniform illumination is a significant drawback. The maximum optical power that the device can produce on the patient's head is 500 mW/cm², necessitating uniform illumination to transfer the maximum possible power. In the helmet approaches this problem has been solved due to the distance between LEDs and head surface 184. A second issue associated with flexible LED strips is the management of temperature due to the energy efficiency of LEDs. Despite the rapid advancement in LED development, challenges of energy efficiency and heat dissipation remain 187, 188.

The problem of uniform lighting could be solved by transistor film 189, wearable fiber 190 and other display approaches 191. An alternative approach utilizing materials based on organic light-emitting diodes (OLEDs) offers numerous advantages 192-194. There are numerous instances where OLEDs have been employed to address uniform lighting issues 195, 196. Moreover, there is an approach that implement fully implantable light emission therapy devices 196-198. OLEDs materials could be a future technology of stimulation specific anatomic area of MLVs due to flexibility and diversity of light emission platforms implementations 196-198.

## 10. Limits and prospects

The recognition of the presence of MLVs in humans and animals, as well as the emergence of reliable markers of the lymphatic endothelium, models of genetically modified mice with fluorescent lymphatics and progress in technologies of neurovisualization, has provided crucial impetus for the development of a new direction in the biophotonics - the *Neuro-Lymphaphotonics*. Modern discoveries have proven that MLVs are "tunnels" for the removal of metabolites and toxins from the brain, which ensures the maintenance of immunity and homeostasis of CNS. Animal studies have shown that dysfunction of MLVs accompanies the development of AD and PD, TBI and oncology. The scientific community is now discussing that the development of technologies for managing the MVL functions will contribute to progress in the treatment of brain diseases. However, this is a difficult task. The MLVs are transparent, thin, with very low lymph flow, which makes their monitoring and, especially, the analysis of peristaltic lymph flow difficult. This review discusses promising directions and solutions for the development of breakthrough phototechnologies for the management of MLVs and the MRI methods for the diagnostic assessment of meningeal lymph flow in both humans and animals. Among the new approaches that may find their application in the future for the assessment of the MLV functions are non-invasive functional near-infrared spectroscopy (fNIRS). The first device in this direction is the glymphometer, based on fNIRS measurement of extracellular fluid in brain tissues 199. The physiological relationship between the glymphatic system and MLVs has not yet been established 134, but it is obvious that fNIRS monitoring of brain drainage have great prospects in the future for analyzing the MLV functions. This review is limited to an analysis of the literature covering the use of fNIRS and other sub-technologies to assess the glymphatic system because we focused on strategies that directly analyze either the MLV morphology or the lymph flow in them, particularly with an emphasis on their clinical application and marketing prospects. However, this first review dedicated to neuro-lymphaphotonics will stimulate the emergence of new ideas and strategies that combine knowledge and technologies to study the lymph/glymphatic system and MLVs, which will open new horizons for future technologies in the treatment of brain diseases.

## Abbreviations

Aβ: amyloid-beta
AD: Alzheimer's disease
ADP: adenosine diphosphate
ATP: adenosine triphosphate
BBB: blood-brain barrier
BLF: brain lymph fluid
CCBE1: calcium-binding EGF domain 1
CNS: central nervous system
COX: cytochrome C oxidase
CSF: cerebral spinal fluid
eNOS: endothelial nitric oxidase
FDA: Food and Drug Administration
fMRI: functional magnetic resonance imaging
fNIRS: functional near-infrared spectroscopy
GBCAs: gadolinium-based contrast agents
ISF: interstitial fluid
LEDs: light-emitting diodes
MLVs: meningeal lymphatic vessels
NIR: near-infrared
NO: nitric oxide
NPLP: nasopharyngeal lymphatic plexus
OLEDs: organic light-emitting diodes
PBM: photobiomodulation
PCB: printed circuit board
PD: Parkinson's disease
PDT: photodynamic therapy
PVSs: perivascular spaces
ROS: reactive oxygen species
TBI: traumatic brain injury
TOF: time-of-flight
